# Targeted next-generation sequencing identifies the disruption of the *SHANK3* and *RYR2* genes in a patient carrying a de novo t(1;22)(q43;q13.3) associated with signs of Phelan-McDermid syndrome

**DOI:** 10.1186/s13039-020-00490-6

**Published:** 2020-06-11

**Authors:** Maria Clara Bonaglia, Sara Bertuzzo, Anna Maria Ciaschini, Giancarlo Discepoli, Lucia Castiglia, Romina Romaniello, Orsetta Zuffardi, Marco Fichera

**Affiliations:** 1 Cytogenetics Laboratory, Scientific Institute, IRCCS Eugenio Medea, Bosisio Parini, Lecco, Italy; 2 Lab. di Genetica Medica SOS Malattie Rare, AOU Ospedali Riuniti Umberto I-G.M.Lancisi-G.Salesi, Ancona, Italy; 3 Oasi Research Institute-IRCCS, Troina, Italy; 4 Neuropsychiatry and Neurorehabilitation Unit, Scientific Institute, IRCCS Eugenio Medea, Bosisio Parini, 23842 Lecco, Italy; 5 Department of Molecular Medicine, University of Pavia, Pavia, Italy; 6 Department of Biomedical and Biotechnological Sciences, Medical Genetics, University of Catania, Catania, Italy

**Keywords:** Phelan-McDermid syndrome, *SHANK3*, *RYR2*, Reciprocal translocation, Balanced rearrangements, Next-generation sequencing, Chromosomal breakpoints, Haploinsufficiency, Cardiac disorder, Genotype-phenotype

## Abstract

**Background:**

It has been known for more than 30 years that balanced translocations, especially if de novo, can associate with congenital malformations and / or neurodevelopmental disorders, following the disruption of a disease gene or its cis-regulatory elements at one or both breakpoints.

**Case presentation:**

We describe a 10-year-old girl with a non-specific neurodevelopmental disorder characterized by moderate intellectual disability (ID), gross motor clumsiness, social and communication deficits. She carries a de novo reciprocal translocation between chromosomes 1q43 and 22q13.3, the latter suggesting the involvement of *SHANK3.* Indeed, its haploinsufficiency associates with Phelan-McDermid Syndrome, whose main symptoms are characterized by global developmental delay and absent or severely delayed expressive speech. A deep molecular approach, including next-generation sequencing of *SHANK3* locus, allowed demonstrating the breakage of *RYR2* and *SHANK3* on the derivative chromosomes 1 and 22 respectively, and the formation of two fusion genes *SHANK3-RYR2* and *RYR2-SHANK3* with concomitant cryptic deletion of 3.6 and 4.1 kilobases at translocation junction of both derivatives chromosomes 22 and 1, respectively.

**Conclusions:**

Although the interruption of *SHANK3* accounts for the patient’s psychomotor retardation and autism-like behavior, we do not exclude that the interruption of *RYR2* may also have a role on her disorder, or result in further pathogenicity in the future. Indeed, *RYR2* that has a well-established role in the etiology of two autosomal dominant adulthood cardiac disorders (#600996 and #604772) is also expressed in the brain (cerebellum, hippocampus, and cerebral cortex) and about half of *RYR2* mutation carriers present late onset primary generalized epilepsy without cardiac arrhythmogenic disorders. Moreover, *RYR2* variants have also been sporadically reported in individuals with early onset schizophrenia or ID, and its constraint values suggest intolerance to loss-of-function. This study not only confirms the usefulness of the molecular mapping of de novo balanced rearrangements in symptomatic individuals, but also underscores the need for long-term clinical evaluation of the patients, for better evaluating the pathogenicity of the chromosomal breakpoints.

## Background

Reciprocal translocations, the incidence of which in unselected infants has been estimated at around 0.2% by conventional cytogenetics [1], are mainly identified in sterile but otherwise healthy males [2] or in couples with repeated abortions or offspring with malformations caused by the unbalanced segregation of the translocation present in a parent [3]. Among those detected in prenatal diagnosis, 6.1% of the de novo ones are at risk of serious congenital anomalies, as estimated by the frequency and outcome of cases detected at amniocentesis, followed over a 10-year period [4]. Interestingly, the risk of neurodevelopment and / or neuropsychiatric disorders after an average 17-year follow-up, for de novo translocations detected in pregnancy with normal first trimester screening / ultrasound, rose to 27% in a cohort of 41 people, a significantly higher frequency than a matched control group [5]. The cloning of several translocation breakpoints and their computational analysis in the context of the threedimensional chromatin conformation have been crucial to reveal how these rearrangements, even when really balanced, may alter the underlying gene expression or its close or long-range regulatory sequences in different ways, including hidden chromothripsis events, eventually leading to the pathogenic condition [5–9]. Moreover, the finding that both translocation breakpoints can fall within gene/regulatory sequences, explains why in many cases patient’s phenotype is complex and difficult to untangle from a clinical perspective. In more rare cases, reciprocal fusion transcripts have been documented both in balanced translocations and inversions [10, 11] but, unlike what happens for acquired rearrangements where the formation of the fusion transcript is an important oncogenic gain-of-function mechanism, in germinal translocations its role is so far less documented [12].

In this regard, the *SHANK3* gene (SH3 and multiple Ankyrin repeat domains; MIM: 606230) was initially associated with PhelanMcDermid syndrome (PMS; MIM # 606232), thanks to the finding that it was interrupted by a reciprocal de novo translocation t(12;22)(q24.1;q13.3), ascertained in a child with all the neurological symptoms of the syndrome [13]. Subsequently, the haploinsufficiency of *SHANK3* was confirmed as the most important factor contributing to the neurobehavioral phenotype of PMS syndrome, characterized by hypotonia, intellectual disability (ID), compromised language (delayed or absent language), behavioral characteristics consistent with disorders of the autistic spectrum and convulsions [14, 15].

Here we describe a girl with moderate psychomotor, behavioral and language deficits, carrying a de novo apparently balanced translocation between chromosome 1 and 22 with a putative *SHANK3* disruption at 22q13.3. A deep molecular approach, including the next generation sequencing of *SHANK3* locus, allowed rapidly demonstrating the formation of two fusion genes *SHANK3-RYR2* at derivative chromosome 22 and *RYR2-SHANK3* at derivative chromosome 1.

*RYR2* (Ryanodine receptor 2) gene, located on 1q43, has a well-established role in stress-induced polymorphic ventricular tachycardia (CPVT1, MIM: 604772) and arrhythmogenic right ventricular dysplasia (ARVC2, MIM:604772). More recently, *RYR2* mutations have been proposed as potential cause of late onset primary generalized epilepsy without cardiac arrhythmogenic disorders [16]. Although the clinical features of our patient fit with the disruption of *SHANK3* gene, we could not exclude a priori that the rewiring of *RYR2* gene due to the translocation t(1;22) may affect our patient’s phenotype later in age.

## Case presentation

### Patient

The girl was born at term by cesarean section to a 32-year-old mother and his 34-year-old father because of breech presentation. Birth weight was 3150 g (50th centile), length 49 cm (50th centile) and cranial circumference (OFC) of 35 cm, (50th centile). Apgar scores 8/9 at 1′/5′, respectively. The perinatal period was unremarkable, apart some difficulties at breast feeding. She achieved motor milestones on time walking without support at the age of 12 months. Her development was unremarkable until the age of 2 years when motor difficulties with frequent falls and stereotypic movements appeared. At the age of 5 years, a moderate psychomotor and language delay became evident. At neurological examination, she showed fine and gross motor clumsiness with unbalance gate. Social skills development was poor with abnormal gaze contact. Brain MRI and EEG recordings tests were normal. Targeted sequence analysis of all exons of *MECP2* did not show any nucleotide variant and FISH test excluded the deletion of one *UBE3A/D15S10* allele (Cytocell). Fragile X DNA test gave normal results. At the age of 6^11/12^ years her weight was 21.5 Kg (25th centile) and height 122.5 cm (50th centile). Unsteady gate, clumsiness and diffuse hypotonia were evident. Social skills were poor and characterized by alternating phases of socialization and isolation. At the last evaluation at the age of 10 years, Wechsler Intelligence Scales (WISC IV) scale score revealed moderate ID (overall IQ = 44) with impairment both in verbal as in performance abilities. Nevertheless, linguistic skills (in particular verbal comprehension) show the better profile. Most difficulties were observed in visuo spatial, visuo perceptive and memory abilities.

**Fig. 1:**
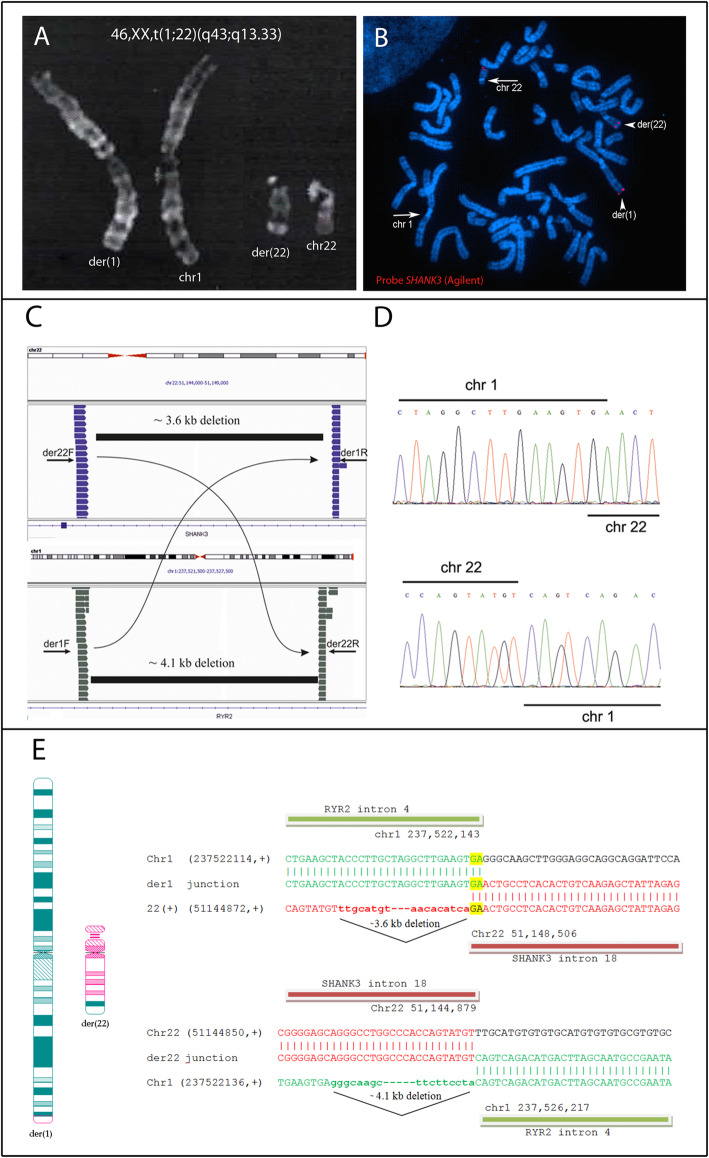
Cytogenetics and molecular results. **a**. Cut-out of normal chromosome 1 and 22, aligned with their homologue translocation derivatives der(1) and der(22) in Q-banding at high resolution (≥550 bands). **b.** Results of metaphase FISH with custom *SHANK3* probe (SureFISH Agilent) showing the normal chromosome 22 (arrow) and both der(1) and der(22) (arrowheads): red signals demonstrate the translocation’s reciprocity. **c.** Split-screen view of the reciprocal translocation (1,22) using the Integrative genomics viewer (IGV). The breakpoint regions for chromosome 22 (top) and for chromosome 1 (bottom) are shown. *Curved lines with arrows* link purple reads on chromosome 22 with their corresponding grey mates mapping on chromosome 1. Black boxes indicate the two subtle deletion regions on chromosome 22 and chromosome 1, respectively. PCR primers used to amplify the breakpoint regions on derivative 1 (der1F - der1R) and on derivative 22 (der22F – der22R) are also shown. **d.** Sanger sequencing defining the breakpoints at the base pair level on derivate 1 (top) and on derivative 22 (bottom). **e.** Karyogram and Sequence alignment of breakpoint junctions confirmed by Sanger sequencing. *(left)* High-resolution Q-banding ideograms of derivative der(1) (*green with a small pink portion*) and der(22) (*pink with a green portion*); *(right)* the junction of der1 (upper) showed a 2 bp (GA) microhomology and the der22 (bottom) junction showed a perfect fusion . Both chromosomes lost bases in the formation of the derivatives 1 and 22, 3.6 Kb on chromosome 22 and 4.1 Kb on chromosome 1

Following normal results chromosome microarray analysis (CMA, Agilent 180 k), Q banding karyotype on metaphase chromosomes revealed an apparently balanced de novo translocation between the distal q arm portion of chromosomes 1 and 22: 46,XX,t(1;22)(q43;q13.3) (Fig. 1a). No 22q bands were visible on der (1). The translocation’s reciprocity was demonstrated by FISH, using the commercially available 22q subtelomeric probe (TelVysion, Vysis), with the chromosome 22 breakpoint within the 22q subtelomeric probe, suggesting a potential disruption of *SHANK3* gene.

## Methods

### FISH investigation

High resolution Q banding metaphase chromosomes were obtained from PHA stimulated blood lymphocytes cultured for 72 h, according to standard protocols [17]. FISH experiment was performed with oligonucleotide based FISH probe, in silico designed to precisely target *SHANK3* gene (AgilentSureFISH, probe size 69,839 kb chr22:51,104,576-51,174,414 GRC37/hg19). The probe *was* synthesized using the company’s high fidelity, oligonucleotide library synthesis (OLS) technology (Agilent Technologies, Santa Clara, CA).

### Next generation sequencing

Genomic DNA was extracted from proband’s and parents’ peripheral blood, obtained after informed consent, using standard procedures. All genomic coordinates are based on the GRCh37/hg19 reference genome assembly.

Agilent Sure Design online tool (https://earray.chem.agilent.com/suredesign/) was used to design enrichment probes targeting the genomic region of *SHANK3* (chr22:51,112,817-51,171,751; hg19). Two thousand four hundred forty-six probes with a coverage of 92% of the target region were obtained. The libraries were prepared using Sure Select QXT Target enrichment kit (Agilent Technologies, Santa Clara, CA). Sample was sequenced on an Illumina Next Seq 500 with 150 bp paired ends reads and a mid output cartridge (Illumina, San Diego, CA). Sequencing reads were aligned against the reference sequence using the Burrows Wheeler Aligner 0.7.7 [18] while base quality score recalibration, duplicate removal, insertions/deletions (indels), indel realignment, and single nucleotide variants’ (SNVs) identifications were carried out using GATK [19] conforming to its “Best Practices” recommendations [20].

The Integrative Genomics Viewer (IGV) was used to visually inspect the quality of read alignment and variant calls. NM_033517.1 and NM_001035.2 were used as references for, respectively, *SHANK3* and *RYR2* exon numbering, according to UCSC genome browser (http://genome.ucsc.edu/cgi-bin/hgTracks?db=hg19).

### Deletions validation and PCR amplification of junction fragment and sanger sequencing

Quantitative polymerase chain reaction (qPCR) assays were performed on DNA from the patient and her parents using SYBR Green and were analyzed on an ABI PRISM 7900HT sequence detection system (Applied Biosystems, Foster City, CA). Long-range (LR) PCR amplification and Sanger sequencing of the deletion breakpoints’ junction were performed with standard protocols. All primer sequences are available from the authors.

### Fusion gene prediction

We predicted the generation of novel fusion protein motifs using Scan Prosite (http://prosite.expasy.org/scanprosite/), as previously reported [21–23]. We evaluated any possible fusion transcripts already collected from various public resources by ChimerDB 2.0 [24].

## Results

### FISH and breakpoints determination

Following routine cytogenetics finding, FISH analysis with a custom *SHANK3* probe (SureFISH Agilent) showed two hybridization signals on the der (1) and der (22) confirming that the reciprocal translocation (1;22) had indeed interrupted the *SHANK3* gene (Fig. 1a, b). To characterize *SHANK3’s* disruption, proband’s DNA was tested by NGS assay targeting the *SHANK3* genomic region, followed by manual inspection of the aligned reads using the IGV software. The search for read pairs that mapped to different chromosomes readily revealed two clusters of discordant read pairs spaced ~ 3.6 kb apart in intron 18 of the *SHANK3* gene, while the corresponding off target mates of each cluster were located approximately 4.1 kb apart in intron 4 of the *RYR2* gene (Ryanodine receptor 2; MIM: 180902), lying on chromosome 1q43 (Fig. 1c).

This allowed us to narrow the translocation breakpoints and to identify two subtle de novo deletions at the chromosome junctions (Fig. 1c), confirmed by an in house qPCR assay (data not shown).

To characterize the breakpoints at nucleotide level, we designed PCR primers flanking the 1:22 and 22:1 junction points, according to discordant read pairs (Fig. 1d). Amplification of genomic DNA using the two couples of primers generated amplicons of ~ 600 bp for derivative chromosome 1 and ~ 550 bp for derivative chromosome 22 that were not present in her parents’ DNA nor in controls. After Sanger sequencing of PCR products and alignment to the reference human genome, we were able to precisely map the derivative chromosome 1 breakpoint at nucleotide 237,522,143 on chromosome 1 and at nucleotide 51,148,506 on chromosome 22 while for the derivative chromosome 22, the breakpoint was located at position 51,144,879 and 237,526,217 on chromosome 22 and chromosome 1, respectively (Fig.1 E). These molecular data allowed us to refine the rearrangement as an unbalanced reciprocal translocation between chromosome 1 and 22.

Based on ISCN2016 nomenclature, the karyotype of this patients was 46,XX,t(1;22)(q43;q13.33)seq[GRCh37]t(1;22)(q43;q13.3)g.[chr1:pter_cen_237522143::chr22:51148506_qter]g.[chr22:pter_cen_51144879::chr1: 237526217_qter] [25].

According to RepeatMasker sequence analysis, the breakpoints were not located within repetitive sequences. Interestingly, a two nucleotide (GA) microhomology was present at derivative chromosome 1 breakpoint, while the junction of derivative 22 showed a perfect fusion (Fig. 1 c).

### Fusion gene prediction

The rearrangement disrupted the structure of *RYR2* and *SHANK3*, probably resulting in a loss of function of both genes, which could not be explored by mRNA expression studies. According to three transcriptomics (HPA, GTEx and FANTOM5) and the consensus datasets, both RYR2 and SHANK3 human proteins are not expressed in patient’s tissues easily accessible, such as blood and skin (https://www.proteinatlas.org). However, it should also be considered that breakpoints at both derivative chromosomes were located within genes which are transcribed in the same orientation, potentially leading to functional fusion transcripts. This hypothesis was tested using the ScanProsite tool (http://prosite.expasy.org/scanprosite/) to predict the reading frames for both fusion genes, i.e. SHANK3-RYR2 at derivative chromosome 22 and RYR2-SHANK3 at derivative chromosome 1. However, both fusion transcripts were expected to be out of the frame (Supplemental Figure S1), which could prevent their translation through sense-mediated RNA decay.

## Discussion and conclusions

Reciprocal chromosome translocations have been crucial to define the locus of Mendelian diseases, first of all those related to the X chromosome. The reports of women suffering from Mendelian pathologies that should have concerned only males, have suggested that other mechanisms, besides a non-random lyonization, could be the basis of the phenomenon. In this sense, the cases of Xp22 / autosomal translocations in females affected by Duchenne Muscular Dystrophy paved the way for, at that time, an unexpected link between Mendelian and chromosomal diseases [26]. These first studies introduced the concept that disease-associated reciprocal translocations were a mine to assign the location of the responsible gene and a network of cytogenetic laboratories has been established to facilitate the identification and mapping of disease-associated balanced chromosomal rearrangements (The Mendelian Cytogenetic Network database, MCNdb). In fact, a causal relationship between a balanced chromosomal abnormality and a congenital anomaly is expected in up to 40% of symptomatic cases [6] and a long-term follow-up study (mean 17 years) estimated that around 27% of de novo translocations considered benign in prenatal diagnosis, hesitates in more or less long times in some neurobehavioural disorder [5]. The study of Halgren et al. [5] indicates that, with the exception of pathological conditions with unequivocal clinical characteristics, the pathogenicity of genes rewired by translocation can be hidden at birth and manifest over the years. The cause of this delayed recognition is probably attributable both to the current limitation in the interpretation of the pathogenicity of some DNA alterations, especially those that concern regulatory regions, and to the manifestation of some conditions only in adulthood or adolescence onwards, as in the case of neuropsychiatric disorders. Moreover, the fact that both translocations breakpoints can interfere with genes whose altered expression may have phenotypic relevance, aggravates the difficulties of both genotype-phenotype interpretation and prognosis, as it occurs in our case.

Indeed, breakpoint sequencing in the present study demonstrated that the reciprocal translocation t(1;2) occurred within two well-known disease-associated genes, *RYR2* and S*HANK3*, via direct gene disruption and concomitant cryptic deletion from 3 to 4 kilobases at translocation junction of both chromosomal derivatives 1 and 22. The clinical condition of our patient, with moderate ID and impairment of both verbal and performance capabilities, behavioral disorders and motor skills deficits is common to many genetic conditions, including the haploinsufficiency of *SHANK3* alone. Accordingly, most of the ailments affecting the patient fit with the disruption of this gene [13, 27, 28]. However, the translocation also disrupted the *RYR2* gene, whose variants have been associated with CPTV1 [29] and ARVC2 [30], two autosomal dominant cardiomyopathies sharing a form of ventricular arrhythmia induced by stress or physical activity that may degenerate to ventricular fibrillation and sudden death. Interestingly, *RYR2* is also expressed in the brain and about half of *RYR2* mutation carriers present with seizures, which are not secondary to arrhythmogenic cardiac dysfunction [31, 32]. *RYR2* variants have also been sporadically reported in individuals with early onset schizophrenia [33] or intellectual disability [34], thus its possible contribution to our patient’s phenotype cannot be excluded a priori. Up to date, almost all the variants identified in the *RYR2-CPTV1* patients are missense changes with clustered location within specific gene domains thus suggesting a gain-of-function pathogenic mechanism consistent with the increased channel activity, indeed demonstrated in some cases [35]. Moreover, a number of duplications interrupting the gene in different portions (ClinVar Long Variants, UCSC GRCh38/hg38) and a recurrent in frame, *Alu*–mediated deletion of exon 3 [36, 37] have been identified in a few CPTV patients, although the pathogenetic mechanism of these deletions has not been clarified. In our patient, the hypothetical fusion transcript *RYR2-SHANK3* is likely to be target by non-sense mediated mRNA decay, in which case the possible pathogenicity of *RYR2* would be caused by its haploinsufficiency. Whatever the possible phenotypic effect of *RYR*2 rupture, our patient at the age of 10 had no evidence of arrhythmias, syncopes or seizures. On the other hand, the average age of the onset of symptoms is between seven and 12 years, although the onset was also reported in the fourth decade of life [GeneReviews: https://www.ncbi.nlm.nih.gov/books/NBK1116/?term=RYR2]. However, as little is known about long term effects of *RYR2* haploinsufficiency in humans, we cannot exclude that *RYR2* haploinsufficiency may represent a risk factor either for neurological or cardiac, late onset clinical manifestations especially in consideration of its constraint metrics (gnomad: https://gnomad.broadinstitute.org/) indicating loss-of-function intolerance [observed / expected: 0.2 (0.16–0.25)].

In conclusion, disruption of the *SHANK3* gene due to reciprocal translocation seems to play the main role in the clinical outcome of our patient. In fact, our proband shows a concordant phenotype with the classical presentation of children of the same age carrying a 22q13.3 linear terminal deletion that involves only the *SHANK3* gene. The breakpoints junction analysis confirmed that seemingly balanced translocation was not balanced at the nucleotide level. The de novo deletion of 3.6 kb in the intron 18 of *SHANK3*, whatever its impact on the patient’s phenotype, would have escaped not only conventional cytogenetic approaches but also the sequencing analysis of the entire exome. Therefore, our study, while confirming the need for molecular mapping of de novo balanced rearrangements in symptomatic individuals, shows at the same time the difficulty of obtaining a detailed correlation and prognosis of how much the breakage of genes intolerant to loss-of-function impacts or will impact the patient’s health.

## Supplementary information

**Additional file 1: Figure S1.** Predicted fusion transcripts. Predicted in-frame fusion genes at translocation junctions to analyze whether the reading frames were conserved by the translocation, as assessed by the ExPASy’s Translation Tool (http://web.expasy.org/translate/) . Open reading frames are highlighted in red while dashed represent premature stop codons. **A)** Fusion transcript RYR2 (NM_001035) - SHANK3 (NM_033517) on derivative chromosome 1 and **B)** Fusion transcript SHANK3 - RYR2 on derivative chromosome 22.

## Data Availability

All primer sequences are available from the authors.

## Abbreviations

ID: Intellectual Disability
PMS: Phelan-McDermid Syndrome
UCSC: University of California Santa Cruz
CNV: Copy number variation
FISH: Fluorescence in situ hybridization
ISCN: International System for Human Cytogenomic Nomenclature
CMA: Chromosome microarray analysis
OMIM: Online Mendelian Inheritance in Man
CPTV1: Ventricular tachycardia, catecholaminergic polymorphic, 1
ARVC2: Arrhythmogenic right ventricular dysplasia 2
MCNdb: The Mendelian Cytogenetic Network database
