# Supplementary material for: Targeted next-generation sequencing identifies the disruption of the SHANK3 and RYR2 genes in a patient carrying a de novo t(1;22)(q43;q13.3) associated with signs of Phelan-McDermid syndrome
Source: Mol Cytogenet. 2020 Jun 11;13:22. doi: 10.1186/s13039-020-00490-6 (PMC7291734; doi:10.1186/s13039-020-00490-6)

**Supplemental Figure 1.** **Predicted fusion transcripts**

Amino acid sequences from predicted in-frame fusion genes at translocation junctions to analyze whether the reading frames were conserved by the translocation, as assessed by the ExPASy’s Translation Tool (http://web.expasy.org/translate/) . Open reading frames are highlighted in red, while red typed M represent methionine amino acids and dashed represent premature stop codons. **A)** Fusion transcript RYR2 (NM_001035) - SHANK3 (NM_033517) on derivative chromosome 1 and **B)** Fusion transcript SHANK3 - RYR2 on derivative chromosome 22.


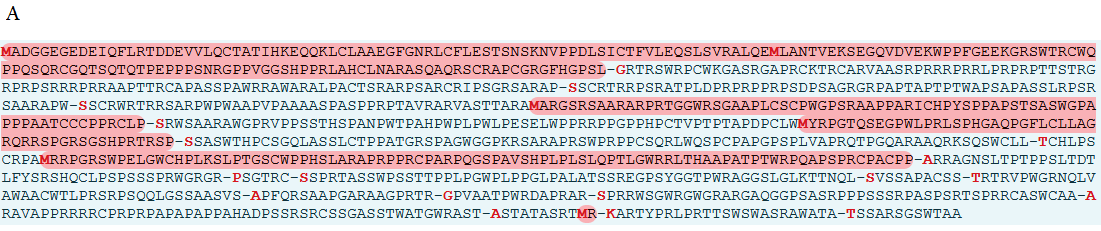


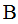


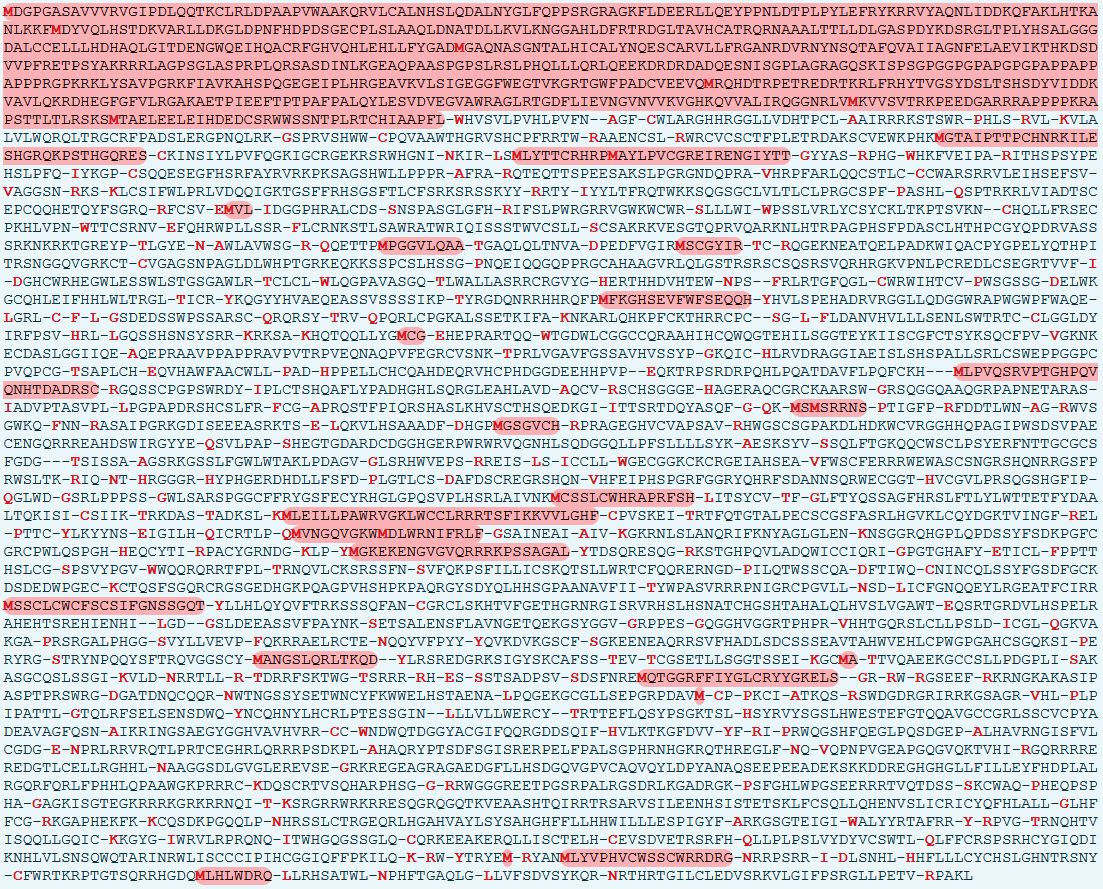

Supplement: Supplementary file 1 — Additional file 1: Figure S1. Predicted fusion transcripts. Predicted in-frame fusion genes at translocation junctions to analyze whether the reading frames were conserved by the translocation, as assessed by the ExPASy’s Translation Tool (http://web.expasy.org/translate/) . Open reading frames are highlighted in red while dashed represent premature stop codons. A) Fusion transcript RYR2 (NM_001035) - SHANK3 (NM_033517) on derivative chromosome 1 and B) Fusion transcript SHANK3 - RYR2 on derivative chromosome 22. [file 13039_2020_490_MOESM1_ESM.docx]
